# A never-ending story: the steadily growing family of the FA and FA-like genes

**DOI:** 10.1590/1678-4685-GMB-2016-0213

**Published:** 2017-05-29

**Authors:** Anna Gueiderikh, Filippo Rosselli, Januario B.C. Neto

**Affiliations:** 1 UMR8200 - CNRS, Équipe labellisée La Ligue contre le Cancer, Villejuif, France; 2 Gustave Roussy Cancer Center, Villejuif, France; 3 Université Paris Saclay, Paris Sud - Orsay, France; 4 Instituto de Biofisica Carlos Chagas Filho, Universidade Federal do Rio de Janeiro, Rio de Janeiro, RJ, Brazil

**Keywords:** DNA repair, leukemia, Fanconi anemia, chromosomal abnormalities

## Abstract

Among the chromosome fragility-associated human syndromes that present cancer predisposition, Fanconi anemia (FA) is unique due to its large genetic heterogeneity. To date, mutations in 21 genes have been associated with an FA or an FA-like clinical and cellular phenotype, whose hallmarks are bone marrow failure, predisposition to acute myeloid leukemia and a cellular and chromosomal hypersensitivity to DNA crosslinking agents exposure. The goal of this review is to trace the history of the identification of FA genes, a history that started in the eighties and is not yet over, as indicated by the cloning of a twenty-first FA gene in 2016.

## Introduction

Fanconi anemia (FA) is a rare human genetic syndrome associated with bone marrow failure (BMF), myelodysplasia (MDS) and a predisposition to acute myeloid leukemia (AML) and head and neck cancer. FA was described in 1927 by the Swiss pediatrician Giuseppe Fanconi, who reported a family with three affected siblings exhibiting anemia and developmental defects (Lobitz and Velleuer, 2006).

The clinical phenotype of FA patients is extremely heterogeneous. Beyond their hematological problems, which constitute the major hallmark of the disease, approximately 70% of these patients present developmental abnormalities, including abnormal radius, absent or supernumerary thumbs, microcephaly, microphthalmia, slow growth rate, café-au-lait spots, skin hyper- and hypo-pigmentation, kidney and urogenital defects, and hypoplasia of the testes. The estimated frequency of the syndrome is 1 in 250,000 - 350,000 live births, with a carrier frequency of approximately 1 in 200 (Rosenberg *et al.*, 2011; Fanconi Anemia Research Fund Inc, 2014).

During the seventies, several groups around the world contributed to the definition of the two major cellular characteristics of the pathology: its particular chromosome fragility and its hypersensitivity to DNA interstrand crosslink (ICL)-inducing agents (Fujiwara and Tatsumi, 1975; Latt *et al.*, 1975; Fornace *et al.*, 1979; Novotna *et al.*, 1979; Ishida and Buchwald, 1982). Indeed, FA cells appear exquisitely sensitive at both the cellular (survival) and chromosomal levels to the exposure to chemicals such as mitomycin C (MMC), diepoxybutane, cis-platinum and photoactivated psoralens. Since it is difficult to distinguish FA patients from individuals suffering from other inherited or idiopathic BMF syndromes on their clinical characteristics alone, the diagnosis of FA is based on the chromosomal response to ICL-inducing agents. Indeed, cytogeneticists score both the basal and induced frequency of chromosome aberrations as well as their subtypes, *i.e.*, tri- and quadriradials, whose presence is quite specific for FA cells (Pinto *et al.*, 2009; Fanconi Anemia Research Fund Inc, 2014).

Based on both the chromosome fragility and the hypersensitivity to exposure to DNA damaging agents, it was quickly suspected that the proteins whose loss of function caused FA must be involved in the DNA damage response and, more specifically, in a DNA repair mechanism. Indeed, although alternative functions associated with each individual or subgroup of FANC proteins exist (Joenje *et al.*, 1981; Rosselli *et al.*, 1994; Fagerlie *et al.*, 2001; Pang *et al.*, 2001; Briot *et al.*, 2008; Pagano *et al.*, 2003, 2012; Zanier *et al.*, 2004; Myers *et al.*, 2011; Justo *et al.*, 2014; Parodi *et al.*, 2015; Sumpter *et al.*, 2016), the well-established “canonical” function of the proteins is to work along a “linear” pathway that addresses replication stresses, assuring the transmission of a stable genome from one cell to the daughters and acting both during DNA replication to cope with stalled replication forks and in G2 and M phases to resolve underreplicated regions before cell division (Ceccaldi *et al.*, 2016; Lopez-Martinez *et al.*, 2016; Michl *et al.*, 2016). How the other, noncanonical functions of the FANC proteins that are involved in cytokine production/response, inflammation (Rosselli *et al.*, 1994; Fagerlie *et al.*, 2001; Pang *et al.*, 2001; Zanier *et al.*, 2004; Briot *et al.*, 2008), mitophagy Sumpter *et al.*, 2016), and oxygen free radical metabolism (Joenje *et al.*, 1981; Pagano *et al.*, 2003; Pagano *et al.*, 2012) as well as the subtle defects in immunity (Myers *et al.*, 2011; Justo *et al.*, 2014; Nguyen *et al.*, 2014; Parodi *et al.*, 2015) impact the clinical and cellular phenotypes of the patients remains a challenge for the future understanding of the pathology.

**Figure 1:**
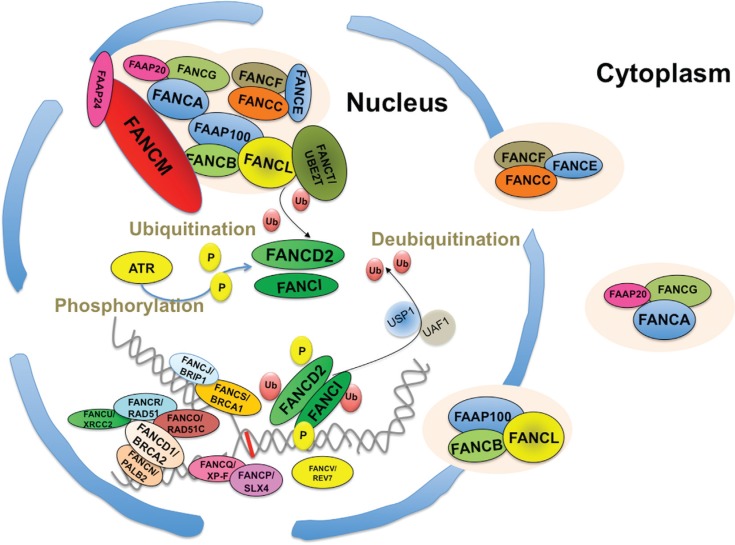
Schematic representation of the subcellular distribution of the FANC proteins, their association and their relocalization in nuclear foci at stalled replication forks. In unstressed conditions, three subcomplexes are present in the nucleus and/or the cytosol: FANCA, FANCG and FAAP20; FANCC, FANCE and FANCF; and FANCB, FANCL and FAAP100. In the presence of DNA damage (the red line represents an interstrand crosslink) that leads to stalled replication forks, all the FANC proteins shuttle into the nucleus to form the FANCcore complex to monoubiquitinate FANCD2 and FANCI, which in turn assemble to subnuclear foci, where they colocalize with several other proteins involved in homologous recombination, including other FANC and FANC-like representatives. The USP1:UAF1 dimer deubiquitinates both FANCD2 and FANCI.

The 21 currently identified *FANC* and *FANC*-like genes (Table 1) encode proteins assembled into three biochemically and functionally defined groups (Wang, 2007). The first group contains several FA and FA-associated proteins (some named FAAPs) that coimmunoprecipitate in the same supramolecular complex. This group is formally called “FANCcore complex” and exhibits the E3 ubiquitin ligase activity responsible for the monoubiquitination of two downstream FANC proteins, FANCD2 and FANCI, which constitute group II. The third group consists of proteins directly involved in DNA metabolism, including structure-specific endonucleases (XP-F and SLX4) and several proteins involved in homologous recombination. Therefore, cells that are defective in genes coding for proteins from group III have normal levels of FANCD2 and FANCI monoubiquitination. The majority of patients (not less than 85%) harbor mutations in genes encoding proteins of the first group. Figure 1 schematically demonstrates the subcellular localization and the assembling pattern of the FANC proteins inside the nucleus in the presence of DNA damage and replication stress, where some of them form subnuclear foci, which can be observed by immunofluorescence microscopic analysis. Briefly, the strongest evidence in the literature supports the presence of three main FANCcore subcomplexes in the cytoplasm and/or in the nucleus, representing triads of proteins - FANCA, FANCG and FAAP20; FANCC, FANCE and FANCF; and FANCB, FANCL and FAAP100. In the nucleus, they assemble into a unique complex on the “cargo” FANCM, which is also associated with the FAAP24 and MHF 1/2 proteins as well as the Bloom-Associated Proteins (BLAPs) (Meetei *et al.*, 2003b; Guo *et al.*, 2009), which are prevented from sliding onto DNA by lesions or a stalled fork. Following an interaction with UBE2T, the complex locally monoubiquitinates FANCD2 and FANCI, a process that requires ATM- and/or ATR- and CHK1-mediated phosphorylation events on both the FANCcore complex proteins and FANCD2 and FANCI. The FANCcore complex monoubiquitinates FANCD2 and FANCI on lysine 561 and 523, respectively. Together, monoubiquitinated FANCD2 and FANCI represent the central link between the upstream FANC proteins (group I or the FANCcore complex) devoid of direct “DNA repair function” and the downstream proteins of group III that are involved in DNA metabolism. Indeed, following their monoubiquitination, FANCD2 and FANCI assemble in chromatin-associated foci, where they colocalize with several FANC and non-FANC proteins directly involved in homologous recombination. The USP1:UAF1 dimer promotes the deubiquitination of both FANCD2 and FANCI, a step necessary to optimally complete the process of DNA repair and for the rescue of stalled replication forks. Several recent reviews have summarized how and when the FANC pathway assumes its role of the guardian of genome integrity (Wang, 2007; Bogliolo and Surralles, 2015; Ceccaldi *et al.*, 2016; Lopez-Martinez *et al.*, 2016; Michl *et al.*, 2016;).

**Table 1:** The 21 currently identified FANC and FANC-like genes.

Complementation group	Extimated frequency	Gene name	Gene alias	Chromosomal position	Protein M.W. (kDa)	Core component	Cloning date	Bona fide FANC gene
A	60-70	*FANCA*		16q24.3	162,7	Yes	1996	Yes
B	rare	*FANCB*	*FAAP95*	Xp22.2	97,7	Yes	2004	Yes
C	10-15	*FANCC*		9q22.3	63,4	Yes	1992	Yes
D1	1-5	*FANCD1*	*BRCA2*	13q12.3	384,2		2002	
D2	1-5	*FANCD2*		3p25.3	164,1		2001	Yes
E	rare	*FANCE*		6p21.3	58,7	Yes	2000	Yes
F	rare	*FANCF*		11p15	42,2	Yes	2000	Yes
G	10-15	*FANCG*	*XRCC9*	9p13.3	68,5	Yes	1998	Yes
I	rare	*FANCI*		15q26.1	149,3	Yes	2007	Yes
J	rare	*FANCJ*	*BACH1; BRIP1*	17q22.2	140,9		2005	?
L	rare	*FANCL*		2p16.1	42,9	Yes	2003	Yes
M	rare	*FANCM*		14q21.2	232,2	Yes	2005	
N	rare	*FANCN*	*PALB2*	16p12.12	131,3		2007	
O	rare	*FANCO*	*RAD51C*	17q22	42,2		2010	
P	rare	*FANCP*	*SLX4*	16p13.3	200		2011	
Q	rare	*FANCQ*	*ERCC4; XPF*	16p13.12	104,5		2013	
R	rare	*FANCR*	*RAD51*	15q15.1	37		2015	
S	rare	*FANCS*	*BRCA1*	17q21	207,7		2015	
T	rare	*FANCT*	*UBE2T*	1q32.1	22,5		2015	Yes
U	rare	*FANCU*	*XRCC2*	7q36.1	31,9		2016	
V	rare	*FANCV*	*REV7*	1p36.22	24,3		2016	Yes

**Figure 2:**
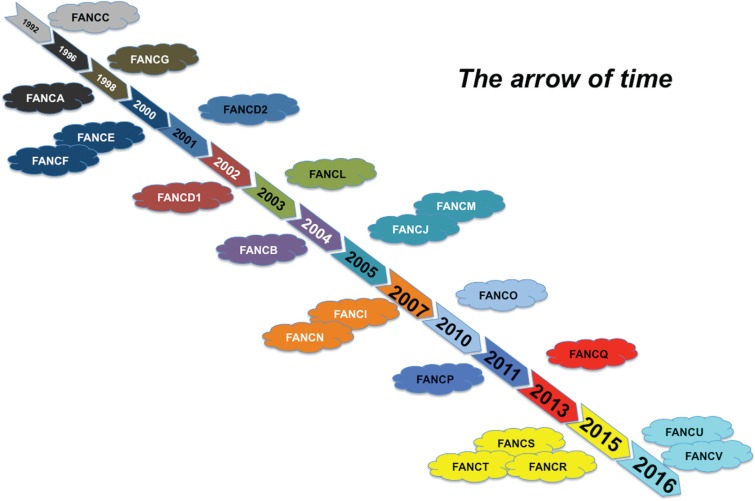
Milestones in the FANC pathway research: a timeline indicating the steps in the discovery of the FANC-BRCA network from the first gene discovered in 1992 to the present.

Here, we sought to retrace the story of the identification of the *FANC* genes (Table 1 and Figure 2). This story exemplifies the evolution of genetics and of molecular biology techniques during the last three decades. Indeed, FA is paradigmatic for several aspects of the human genetics field.

It is important to note that in recent years, the criteria to be considered as a “bona fide FA gene” have become more stringent and are now based on the clinical phenotype. Indeed, whereas the loss of function of all the identified genes leads to the primary FA cellular characteristics, including the ICL hypersensitivity and chromosome fragility, the clinical traits of some patients fail to reach the canonical features of the FA syndrome, namely BMF and the MDS. The genes mutated in those patients are now excluded from the “bona fide FA gene” group and are considered as “FA-like genes” (Bofliolo and Surralles, 2015). Nevertheless, the number of *bona fide FANC* and *FANC*-like genes continues to grow and it is unlikely to stop anytime soon.

## History of the identification of *FANC* genes

### One disease, many genes

The existence of genetic heterogeneity in FA was demonstrated at the beginning of the eighties by the pioneering work of the groups of Manuel Buchwald and Karl Sperling, which used a cell fusion approach (Zakrzewski and Sperling, 1980; Duckworth-Rysiecki *et al.*, 1985). In particular, Duckworth-Risiecky and collaborators reported the existence of two FA complementation groups: A and non-A. The complementation analysis was based on the rescue of the cellular and chromosomal sensitivity to MMC exposure in the hybrid cells compared to the two cell lines fused to obtain the hybrid (Duckworth-Rysiecki *et al.*, 1985).

### The nineties

Seven years later, in 1992, the Buchwald group published two seminal manuscripts, with the first recognizing that the non-A group was heterogeneous, assembling three complementation groups: B, C and D (Strathdee *et al.*, 1992a). The second manuscript reported the cloning of the first *FANC* gene, *FANCC* (Strathdee *et al.*, 1992b). To identify the mutated gene, Strathdee and collaborators followed a functional complementation strategy. They transfected an EBV-based cDNA expression library into the HSC-536 B-lymphoblastoid cell line (previously assigned to the FA-C complementation group) and isolated three overlapping cDNA sequences able to independently complement the huge cellular and chromosomal hypersensitivity to MMC of the transfected cells. The proband carried a mutation of maternal origin that changed leucine 544 to a proline (L544P), a modification predicted to disrupt an alpha helical secondary structure of the protein (Strathdee *et al.*, 1992b). Initially elusive, the mutation affecting the allele of paternal origin, a deletion of 327 bp resulting in the removal of exons 1 and 2, was identified some years later (Parker *et al.*, 1998).

A new complementation group was added to the list in 1995. By analyzing 13 unrelated FA patients, Joenje *et al.* (1995) identified a fifth FA complementation group, named FA-E. The following year, two groups cloned *FANCA,* the most frequently mutated *FANC* gene, using two alternative strategies. One group identified the gene by the same functional complementation approach as used by the Buchwald group (Lo Ten Foe et al., 1996). Alternatively a consortium of several laboratories working to identify breast cancer susceptibility genes adopted the chromosome walking strategy (Fanconi anaemia/Breast Cancer, 1996) after the localization of a putative FANCA gene in the q24.3 region of the chromosome 16 (Pronk *et al.*, 1995).

In 1997, the number of complementation groups established by the original cell fusion approach grew again, totaling 8: A to G (Joenje *et al.*, 1997). In 1998, de Winter *et al.* (1998) used functional complementation to isolate a cDNA able to rescue the MMC hypersensitivity of a standard FA-G cell line. The identified sequence, renamed *FANCG*, was similar to that of a gene cloned one year before on the basis of its capability to complement the MMC hypersensitivity of a CHO UV40 mutant clone called XRCC9 (Liu *et al.*, 1997).

### The 2000s

The third millennium opened with the cloning of two new *FANC* genes. Still using the functional complementation cloning approach, de Winter and collaborators identified the genes whose loss of function was associated with the FA complementation groups E and F, *i.e.*, *FANCE*, (de Winter *et al.*, 2000a), previously mapped on chromosome 6 (Waisfisz *et al.*, 1999), and *FANCF* (de Winter *et al.*, 2000b).

In 2001, Timmers and collaborators reported that the cell lines originally categorized in the FA complementation group D (FA-D) could be separated into two groups, named D1 and D2, and they identified the gene mutated in the D2 group, *FANCD2*, by positional cloning and chromosome transfer, owing to the previous identification of the gene on chromosome 3p (Whitney *et al.*, 1995; Hejna *et al.*, 2000; Timmers *et al.*, 2001).

The next year, D'Andrea and colleagues decided to test the hypothesis that the inactivation of *BRCA1* and *BRCA2*, the most famous and frequently mutated genes in familial predisposition to breast cancer and whose loss of function results in a cellular phenotype similar to that described for FA, could also be involved in FA. A systematic sequencing of *BRCA1* and *BRCA2* was performed in several FA cell lines that belonged to complementation groups without an assigned gene. This “target gene” approach allowed the identification of biallelic variations in *BRCA2* in the FA-D1 standard cell line HSC-62. The variants were successively validated as inactivating mutations (Howlett *et al.*, 2002), assigning *BRCA2* to the list of *FANC* genes as *FANCD1*. Howlett and collaborators also identified some variants of *BRCA1* by examining the HSC230 cell line, the FA-B standard cell line. However, these variants failed to be confirmed as *bona fide* inactivating mutations.

In 2003, a big step forward in the genetics of FA was achieved thanks to the work of Meetei *et al.* (2003b) in the Weidong Wang laboratory. They purified a BLM-associated supramolecular complex containing two salt-concentration-dependent separable groups of proteins as follows: the BLM-associated proteins (BLMAPs) and Fanconi anemia-associated polypeptides (FAAPs). Mass spectrometry analysis of the isolated FAAPs identified some of the known FANC proteins (FANCA, FANCC, FANCE, FANCF, and FANCG), and the unknown components were identified as FAAP43, 90/95, 100, and 250/300 on the basis of their molecular mass. The biochemical approach of the Wang group allowed the cloning of new *FANC* genes by a “protein to gene” walking route. Indeed, after the identification of the amino acid composition of the FAAPs, the authors were able to match this to the sequence of each corresponding gene, to look for mutations in cells from FA patients and/or to determine the gene function(s) by analyzing the phenotypic consequences of the engineered inactivation of these genes in model cells or mice. Moreover, the work of Meetei and collaborators also provided the first indication that at least some of the FANC proteins work together inside a molecular complex.

The same year, Meetei *et al.* (2003a) identified FAAP43 as the PHD finger protein 9 coding gene, or *PHF9*. It was known that the inactivation of the mouse homolog of *PHF9, Pog* (for proliferation of germ cells), resulted in infertility and the MMC hypersensitivity of bone marrow cells, the two more consistent features presented by the already obtained FA mouse models. To definitively validate that PHF9 belonged to the *FANC* gene family, Meetei and coworkers identified inactivating PHF9 mutations in a cell line, EUFA868, isolated from a patient not previously assigned to a complementation group. The EUFA868 cell line used to clone *PHF9* was assigned to FA-L and *PHF9* was also named *FANCL* (Meetei *et al.*, 2003a). *PHF9/FANCL* encodes the ubiquitin E3 ligase of the FANCcore complex, which mediates the FANCD2 and FANCI monoubiquitination (Meetei *et al.*, 2004b). A second FA patient bearing a mutation in *FANCL* was identified six years later (Ali *et al.*, 2009).

In 2004, Meetei *et al.* (2004a) identified the coding sequence of FAAP95 as being similar to the one named FLJ34064, a sequence localized on the X chromosome. Mutations in FLJ34064 were found in several FA cell lines, including the HSC-230 cell line, the standard for FA complementation group B. Thus, *FAAP95* was renamed *FANCB*. *FANCB* is the only known *FANC* gene localized on the X chromosome. Consequently, its inactivation affects only males. *FANCB* is silenced via the methylation of its promoter on the X chromosome that undergoes inactivation during embryogenesis. Since the X-inactivation is stochastic, *i.e.*, it affects either the paternal or the maternal X chromosome randomly, it is expected that the expression of a gene subjected to inactivation will exhibit mosaicism. In the case of *FANCB*+/- female carriers, the large majority of the lymphocytes and fibroblasts of the body express the WT gene, suggesting that the cells that express the mutated gene are counterselected, probably due to their growth difficulties, and are rapidly lost (Meetei *et al.*, 2004a).

In 2005, Meetei *et al.* (2005) identified FAAP250 as KIAA1596, a human protein with sequence similarities to DNA repair proteins, including the yeast MPH1 and the human ERCC4/XP-F. siRNA-mediated depletion of KIAA1596 in cellular models affected FANCD2 monoubiquitination and increased MMC sensitivity, arguing for the assignment of FAAP250 to the *FANC* gene family. Mutations were then identified in an FA patient who was not assigned to a known complementation group. The gene was called *FANCM*, and the new complementation group FA-M. Nevertheless, the attempts to complement the MMC hypersensitivity of cell lines derived from the patient by transfection of the wild-type *FANCM* cDNA failed. Surprisingly, it was demonstrated that he also carried biallelic mutations in *FANCA.* Therefore, even if the loss of function of FANCM by targeted mutagenesis in mice or by siRNA-mediated depletion in human cells results in an FA-like cellular phenotype and in spite of its interaction with several other FANC proteins, the lack of patients with the major features of FA and only the loss of *FANCM* function impedes the assignment of the protein to the group of the “bona fide” *FANC* genes (Meetei *et al.*, 2005; Singh *et al.*, 2009).

In the same year, 2005, three groups identified the gene mutated in FA-J cells and named it *FANCJ* (Levitus *et al.*, 2005; Levran *et al.*, 2005; Litman *et al.*, 2005). Following unsuccessful attempts to identify the mutated gene by a complementation cloning strategy, Levitus *et al.* (2005) opted for a positional cloning strategy and identified in eight FA-J cell lines several pathogenic mutations in the gene encoding the DEAH-box DNA helicase and binding partner of BRCA1 BRIP1/BACH1 (BRCA1-Immunoprecipitated Protein 1/BRCA1-associated C-terminal helicase-1), which was previously cloned by Cantor *et al.* (2001). Using a genome-wide scan, Levran *et al.* (2005) identified a homozygous region on chromosome 17q23 in which there were two interesting candidate genes: *RAD51C* and *BRIP1.* Inactivating mutations were found only in *BRIP1/BACH1*. Finally, Litman *et al.* (2005) identified *BRCA1/BACH1* mutations in two families that were associated with an early onset of breast cancer and found the same recurrent nonsense mutation, the R798X mutation in exon 17, in both the breast cancer and FA-J families. This mutation affected the helicase domain of the protein, and since it was identified in people of different ethnic origin, it likely represents a hot spot of mutation or an inactivating event that remains compatible with survival (Levitus *et al.*, 2005). Thus, *BRIP1/BACH1* is also called *FANCJ*.

In 2007, following alternative approaches, three independent groups cloned the 13th *FANC* gene, *FANCI*, which is the paralog of *FANCD2* (Dorsman *et al.*, 2007; Meijer, 2007; Sims *et al.*, 2007; Smogorzewska *et al.*, 2007). *FANCI* was identified by a linkage analysis approach (Dorsman *et al.*, 2007), by a bioinformatical screening for *FANCD2* homologs (Sims *et al.*, 2007) and by a proteomic search for ATM and ATR targets (Smogorzewska *et al.*, 2007). It was described as the gene mutated in cells belonging to FA complementation group I.

Also in 2007, the 14th *FANC* gene was cloned and assigned to FA-N, a previously unrecognized FA complementation group (Reid *et al.*, 2007; Xia *et al.*, 2007). The identified gene, *FANCN,* was known to encode PALB2, isolated by immunoprecipitation one year prior as the Partner and Localizer of BRCA2 (Xia *et al.*, 2006). Reid *et al.* (2007) followed a candidate gene approach, sequencing 82 FA patients with unelucidated genetic causes and identified mutations inactivating PALB2 in 7 individuals belonging to independent families. By Western blot analysis, Xia and collaborators noticed the lack of a full-length PALB2 in an unassigned FA cell line. Subsequent DNA sequencing allowed the identification of the inactivating mutations in *PALB2* (Xia *et al.*, 2007).

In 2010, aiming to identify the gene responsible for the pathology in a Pakistani family with FA by a genome-wide mapping approach, Vaz *et al.* (2010) identified a homozygous mutation in the *RAD51C* gene. Successively, *in vitro* functional studies showed that the identified mutation resulted in the loss of RAD51 focus formation in response to DNA damage, a defect that could be rescued by the ectopic expression of wild-type *RAD51C*. On this basis, the authors proposed to assign the acronym *FANCO* to *RAD51C*. *RAD51C* is also recognized as a gene associated with breast and ovarian cancer predisposition (Somyajit *et al.*, 2012).

In 2011, several groups focused their work on *SLX4*, a gene previously identified in yeast and flies as well as in humans and involved in the cellular response to DNA ICLs (Mullen *et al.*, 2001; Wu *et al.*, 2004; Lee *et al.*, 2005; Fekairi *et al.*, 2009; Munoz *et al.*, 2009; Svendsen *et al.*, 2009). Kim *et al.* (2011) and Stoepker *et al.* (2011) decided to sequence the *SLX4* gene in several patients with an FA-like phenotype, who until that time had not been assigned to any of the sixteen known complementation FA groups. They successfully identified some patients with mutations in the SLX4 coding sequence. On the other hand, Crossan *et al.* (2011) found that Slx4-null mice recapitulated the features of FA. Thus, *SXL4* was also named *FANCP*. It codes for a structure-specific endonuclease that can be found in a complex with XP-F/ERCC1 and MUS81/EME1, proteins involved in protecting the genome during S and M phases.

Surprisingly, in 2013, inactivating mutations in *ERCC4/XP-F*, whose loss of function was previously associated with the skin cancer predisposition syndrome Xeroderma pigmentosum complementation group F, were also identified to be associated with an FA-like phenotype by whole-exome and Sanger sequencing of the DNA of unclassified FA individuals (Bogliolo *et al.*, 2013). *ERCC4/XP-F* was thus renamed *FANCQ*. Analyses of the consequences of the identified mutations clearly demonstrated that compared to the NER-associated mutations, these mutations altered different regions, affecting an alternative function of the protein (Bogliolo *et al.*, 2013). The association of *XP-F* mutations with an FA phenotype was successfully validated by an independent analysis (Kashiyama *et al.*, 2013). Therefore, genetic defects in the structure-specific endonuclease XP-F/ERCC1 can result in xeroderma pigmentosum, Cockayne syndrome, Fanconi anemia, XFE progeria and cerebro-oculo-facio-skeletal syndrome (Manandhar *et al.*, 2015).

Three important articles were published in 2015. Using whole-genome sequencing, Ameziane *et al.* (2015) identified a heterozygous dominant negative *de novo* mutation in the RAD51-encoding gene in an atypical FA patient and suggested adding the acronym *FANCR* to the major homologous recombination player known to play a role in both the resistance (if overexpressed) or sensitivity (when mutated or underexpressed) of cancer cells to radio- and chemotherapies and whose haploinsufficiency is involved in the congenital mirror movement neurological disorder (Depienne *et al.*, 2012).

The second 2015 article was published by Sawyer *et al.* (2015). They described a patient with a complex FA-like phenotype carrying hereditary biallelic mutations in *BRCA1.* Indeed, the patient was identified in 2013 (Domchek *et al.*, 2013), but the clinical phenotype of the patient was ascertained definitively only two years later. Therefore, thirteen years after the discovery that the gene for FA-D1 is *BRCA2, BRCA1* has likewise obtained the acronym *FANCS*.

Likewise, Hira *et al.* (2015) described one individual harboring the classical cellular features and symptoms of FA and bearing biallelic mutations in the gene coding for an E2 ubiquitin-conjugating enzyme, UBE2T. UBE2T was originally identified by Zhang *et al.* (2000) and recognized as the principal ubiquitin E2 ligase of the FANCcore complex by Machida *et al.* (2006). *UBE2T* was, indeed, renamed *FANCT*.

In 2016, *XRCC2* was identified as *FANCU* (Park *et al.*, 2016). However, the patient who carried the *XRCC2* mutations failed to show bone marrow failure. XRCC2 belongs to a group of RAD51 paralogs, which includes RAD51B, C and D. Therefore, with *XRCC2/FANCU*, the list of genes of which inactivating mutations could be associated with an FA-like phenotype and with breast cancer predisposition and/or homologous recombination now includes *BRCA2, BRCA1, PALB2, BRIP1/BACH1, RAD51* and *RAD51C.*

Also in 2016, the 21st and last (but probably not for long) FA or FA-like gene was cloned (Bluteau *et al.*, 2016). These authors identified a child with severe BMF harboring biallelic inactivating mutations in the gene encoding the translesion DNA synthesis (TLS) protein subunit REV7 (also known as MAD2L2), which was named *FANCV*. FANCV plays a central role in the bypass of the unhooked ICL downstream FANCD2/FANCI, allowing the progression of the process that leads to the HR-mediated rescue of the DNA replication impeded by the stall and collapse of an ongoing replication fork at the DNA lesion.

## What next?

While 21 *FANC* genes have been identified and although the alphabet is near its end, the story is probably far from being finished. Obviously, patients bearing mutations in new genes are expected to be extremely rare. However, just looking at FANCM and the FANCcore complex partners, no fewer than five genes could claim the title of “*FANC* or *FANC-like* gene”: *FAAP20, FAAP24* and FAAP*100*, *MHF1* and *MHF2*. The depletion or deletion of these proteins results in an FA-like cellular phenotype and mouse mutants, when derived, present a phenotype similar to that of the majority of the FANC-KO mouse models. However, because patients with these mutations are rare, the formal attribution of a FANC acronym to previous genes is currently impossible. Loss-of-function mutations of USP1, the FANCD2/FANCI deubiquitinase, result in an FA-like phenotype in a mouse model and in human cells. Indeed, to be unable to monoubiquitinate FANCD2 or to have FANCD2 constitutively monoubiquitinated represents a similarly poor fate for a cell. However, again, no patient bearing USP1 mutations and presenting the FA clinical symptoms have yet been identified. Moreover, the loss of function of the other components of the structure-specific endonuclease heterodimers MUS81-EME1 (and possibly also EME2), XPF-ERCC1 and SLX4-SLX1 could also be associated with patients with an FA-like phenotype, although, for the moment, their potential mutations are associated with either lethal or extremely strong clinical phenotypes that probably preclude the possibility of their assignment to FA. Additionally, mutations in several other known HR-associated proteins could also result in an FA or an FA-like phenotype in some rare families.

In conclusion, the story of the identificatioin of the FANC genes allows to appreciate the evolution of the genetic and molecular techniques to identify disease-associated genes and to better define their lonks with the pathological traits. Also, considering the divergent clinical phenotypes associated with the loss of function of the gene products involved in the resistance to DNA crosslinking agents, it seems important to stress again that not all the ICL-repair proteins can nowadays claim to be members of the *FANC* gene group, even if they are involved in the FANC pathway. In the future, the upper part of the FANC pathway, consisting of the FANCcore complex-encoding genes FANCD2/FANCI and some of the proteins of the third group (FANCQ, FANCV), will probably be considered separately from the bottom part, whose associated gene products are involved in homologous recombination biochemistry and in breast and ovarian cancer predisposition, for which biallelic germinal inactivation results in strong clinical phenotypes.

## References

[B1] Ali AM, Kirby M, Jansen M, Lach FP, Schulte J, Singh TR, Batish SD, Auerbach AD, Williams DA, Meetei AR (2009). Identification and characterization of mutations in FANCL gene: A
second case of Fanconi anemia belonging to FA-L complementation
group. Hum Mutat.

[B2] Ameziane N, May P, Haitjema A, van de Vrugt HJ, van Rossum-Fikkert SE, Ristic D, Williams GJ, Balk J, Rockx D, Li H (2015). A novel Fanconi anaemia subtype associated with a dominant-negative
mutation in RAD51. Nat Commun.

[B3] Bluteau D, Masliah-Planchon J, Clairmont C, Rousseau A, Ceccaldi R, Dubois d'Enghien C, Bluteau O, Cuccuini W, Gachet S (2016). Biallelic inactivation of REV7 is associated with Fanconi
anemia. J Clin Invest.

[B4] Bogliolo M, Schuster B, Stoepker C, Derkunt B, Su Y, Raams A, Trujillo JP, Minguillon J, Ramirez MJ, Pujol R (2013). Mutations in ERCC4, encoding the DNA-repair endonuclease XPF, cause
Fanconi anemia. Am J Hum Genet.

[B5] Bogliolo M, Surralles J (2015). Fanconi anemia: A model disease for studies on human genetics and
advanced therapeutics. Curr Opin Genet Dev.

[B6] Briot D, Mace-Aime G, Subra F, Rosselli F (2008). Aberrant activation of stress-response pathways leads to TNF-alpha
oversecretion in Fanconi anemia. Blood.

[B7] Cantor SB, Bell DW, Ganesan S, Kass EM, Drapkin R, Grossman S, Wahrer DC, Sgroi DC, Lane WS, Haber DA (2001). BACH1, a novel helicase-like protein, interacts directly with BRCA1
and contributes to its DNA repair function. Cell.

[B8] Ceccaldi R, Sarangi P, D'Andrea AD (2016). The Fanconi anaemia pathway: New players and new
functions. Nat Rev Mol Cell Biol.

[B9] Crossan GP, van der Weyden L, Rosado IV, Langevin F, Gaillard PH, McIntyre RE, Gallagher F, Kettunen MI, Lewis DY, Sanger Mouse Genetics Project (2011). Disruption of mouse Slx4, a regulator of structure-specific nucleases,
phenocopies Fanconi anemia. Nat Genet.

[B10] de Winter JP, Waisfisz Q, Rooimans MA, van Berkel CG, Bosnoyan-Collins L, Alon N, Carreau M, Bender O, Demuth I, Schindler D (1998). The Fanconi anaemia group G gene FANCG is identical with
XRCC9. Nat Genet.

[B11] de Winter JP, Leveille F, van Berkel CG, Rooimans MA, van Der Weel L, Steltenpool J, Demuth I, Morgan NV, Alon N, Bosnoyan-Collins L (2000a). Isolation of a cDNA representing the Fanconi anemia complementation
group E gene. Am J Hum Genet.

[B12] de Winter JP, Rooimans MA, van Der Weel L, van Berkel CG, Alon N, Bosnoyan-Collins L, de Groot J, Zhi Y, Waisfisz Q, Pronk JC (2000b). The Fanconi anaemia gene FANCF encodes a novel protein with homology
to ROM. Nat Genet.

[B13] Depienne C, Bouteiller D, Meneret A, Billot S, Groppa S, Klebe S, Charbonnier-Beaupel F, Corvol JC, Saraiva JP, Brueggemann N (2012). RAD51 haploinsufficiency causes congenital mirror movements in
humans. Am J Hum Genet.

[B14] Domchek SM, Tang J, Stopfer J, Lilli DR, Hamel N, Tischkowitz M, Monteiro AN, Messick TE, Powers J, Yonker A (2013). Biallelic deleterious BRCA1 mutations in a woman with early-onset
ovarian cancer. Cancer Discov.

[B15] Dorsman JC, Levitus M, Rockx D, Rooimans MA, Oostra AB, Haitjema A, Bakker ST, Steltenpool J, Schuler D, Mohan S (2007). Identification of the Fanconi anemia complementation group I gene,
FANCI. Cell Oncol.

[B16] Duckworth-Rysiecki G, Cornish K, Clarke CA, Buchwald M (1985). Identification of two complementation groups in Fanconi
anemia. Somatic Cell Mol Genet.

[B17] Fagerlie S, Lensch MW, Pang Q, Bagby GC (2001). The Fanconi anemia group C gene product: Signaling functions in
hematopoietic cells. Exp Hematol.

[B18] Fanconi Anaemia Research Fund Inc and Breast Cancer (1996). Positional cloning of the Fanconi anaemia group A gene. Nat Genet.

[B19] Fanconi Anemia Research Fund Inc (2014). Fanconi anemia: Guidelines for Diagnosis and Management.

[B20] Fekairi S, Scaglione S, Chahwan C, Taylor ER, Tissier A, Coulon S, Dong MQ, Ruse C, Yates JR, Russell P (2009). Human SLX4 is a Holliday junction resolvase subunit that binds
multiple DNA repair/recombination endonucleases. Cell.

[B21] Fornace AJ, Little JB, Weichselbaum RR (1979). DNA repair in a Fanconi's anemia fibroblast cell
strain. Biochim Biophys Acta.

[B22] Fujiwara Y, Tatsumi M (1975). Repair of mitomycin C damage to DNA in mammalian cells and its
impairment in Fanconi's anemia cells. Biochem Biophys Res Commun.

[B23] Guo R, Xu D, Wang W (2009). Identification and analysis of new proteins involved in the DNA damage
response network of Fanconi anemia and Bloom syndrome. Methods.

[B24] Hejna JA, Timmers CD, Reifsteck C, Bruun DA, Lucas LW, Jakobs PM, Toth-Fejel S, Unsworth N, Clemens SL, Garcia DK (2000). Localization of the Fanconi anemia complementation group D gene to a
200-kb region on chromosome 3p25.3. Am J Hum Genet.

[B25] Hira A, Yoshida K, Sato K, Okuno Y, Shiraishi Y, Chiba K, Tanaka H, Miyano S, Shimamoto A, Tahara H (2015). Mutations in the gene encoding the E2 conjugating enzyme UBE2T cause
Fanconi anemia. Am J Hum Genet.

[B26] Howlett NG, Taniguchi T, Olson S, Cox B, Waisfisz Q, De Die-Smulders C, Persky N, Grompe M, Joenje H, Pals G (2002). Biallelic inactivation of BRCA2 in Fanconi anemia. Science.

[B27] Ishida R, Buchwald M (1982). Susceptibility of Fanconi's anemia lymphoblasts to DNA-cross-linking
and alkylating agents. Cancer Res.

[B28] Joenje H, Arwert F, Eriksson AW, de Koning H, Oostra AB (1981). Oxygen-dependence of chromosomal aberrations in Fanconi's
anaemia. Nature.

[B29] Joenje H, Lo Ten Foe JR, Oostra AB, van Berkel CG, Rooimans MA, Schroeder-Kurth T, Wegner RD, Gille JJ, Buchwald M, Arwert F (1995). Classification of Fanconi anemia patients by complementation analysis:
Evidence for a fifth genetic subtype. Blood.

[B30] Joenje H, Oostra AB, Wijker M, di Summa FM, van Berkel CG, Rooimans MA, Ebell W, van Weel M, Pronk JC, Buchwald M (1997). Evidence for at least eight Fanconi anemia genes. Am J Hum Genet.

[B31] Justo GA, Bitencourt MA, Pasquini R, Castelo-Branco MT, Almeida-Oliveira A, Diamond HR, Rumjanek VM (2014). Immune status of Fanconi anemia patients: Decrease in T CD8 and
CD56dim CD16+ NK lymphocytes. Ann Hematol.

[B32] Kashiyama K, Nakazawa Y, Pilz DT, Guo C, Shimada M, Sasaki K, Fawcett H, Wing JF, Lewin SO, Carr L (2013). Malfunction of nuclease ERCC1-XPF results in diverse clinical
manifestations and causes Cockayne syndrome, xeroderma pigmentosum, and Fanconi
anemia. Am J Hum Genet.

[B33] Kim Y, Lach FP, Desetty R, Hanenberg H, Auerbach AD, Smogorzewska A (2011). Mutations of the SLX4 gene in Fanconi anemia. Nat Genet.

[B34] Latt SA, Stetten G, Juergens LA, Buchanan GR, Gerald PS (1975). Induction by alkylating agents of sister chromatid exchanges and
chromatid breaks in Fanconi's anemia. Proc Natl Acad Sci U S A.

[B35] Lee W, St Onge RP, Proctor M, Flaherty P, Jordan MI, Arkin AP, Davis RW, Nislow C, Giaever G (2005). Genome-wide requirements for resistance to functionally distinct
DNA-damaging agents. PLoS Genet.

[B36] Levitus M, Waisfisz Q, Godthelp BC, de Vries Y, Hussain S, Wiegant WW, Elghalbzouri-Maghrani E, Steltenpool J, Rooimans MA, Pals G (2005). The DNA helicase BRIP1 is defective in Fanconi anemia complementation
group J. Nat Genet.

[B37] Levran O, Attwooll C, Henry RT, Milton KL, Neveling K, Rio P, Batish SD, Kalb R, Velleuer E, Barral S (2005). The BRCA1-interacting helicase BRIP1 is deficient in Fanconi
anemia. Nat Genet.

[B38] Litman R, Peng M, Jin Z, Zhang F, Zhang J, Powell S, Andreassen PR, Cantor SB (2005). BACH1 is critical for homologous recombination and appears to be the
Fanconi anemia gene product FANCJ. Cancer Cell.

[B39] Liu N, Lamerdin JE, Tucker JD, Zhou ZQ, Walter CA, Albala JS, Busch DB, Thompson LH (1997). The human XRCC9 gene corrects chromosomal instability and mutagen
sensitivities in CHO UV40 cells. Proc Natl Acad Sci U S A.

[B40] Lo Ten Foe JR, Rooimans MA, Bosnoyan-Collins L, Alon N, Wijker M, Parker L, Lightfoot J, Carreau M, Callen DF, Savoia A (1996). Expression cloning of a cDNA for the major Fanconi anaemia gene,
FAA. Nat Genet.

[B41] Lobitz S, Velleuer E (2006). Guido Fanconi (1892-1979): A jack of all trades. Nat Rev Cancer.

[B42] Lopez-Martinez D, Liang CC, Cohn MA (2016). Cellular response to DNA interstrand crosslinks: The Fanconi anemia
pathway. Cell Mol Life Sci.

[B43] Machida YJ, Machida Y, Chen Y, Gurtan AM, Kupfer GM, D'Andrea AD, Dutta A (2006). UBE2T is the E2 in the Fanconi anemia pathway and undergoes negative
autoregulation. Mol Cell.

[B44] Manandhar M, Boulware KS, Wood RD (2015). The ERCC1 and ERCC4 (XPF) genes and gene products. Gene.

[B45] Meetei AR, de Winter JP, Medhurst AL, Wallisch M, Waisfisz Q, van de Vrugt HJ, Oostra AB, Yan Z, Ling C, Bishop CE (2003a). A novel ubiquitin ligase is deficient in Fanconi
anemia. Nat Genet.

[B46] Meetei AR, Sechi S, Wallisch M, Yang D, Young MK, Joenje H, Hoatlin ME, Wang W (2003b). A multiprotein nuclear complex connects Fanconi anemia and Bloom
syndrome. Mol Cell Biol.

[B47] Meetei AR, Levitus M, Xue Y, Medhurst AL, Zwaan M, Ling C, Rooimans MA, Bier P, Hoatlin M, Pals G (2004a). X-linked inheritance of Fanconi anemia complementation group
B. Nat Genet.

[B48] Meetei AR, Yan Z, Wang W (2004b). FANCL replaces BRCA1 as the likely ubiquitin ligase responsible for
FANCD2 monoubiquitination. Cell Cycle.

[B49] Meetei AR, Medhurst AL, Ling C, Xue Y, Singh TR, Bier P, Steltenpool J, Stone S, Dokal I, Mathew CG (2005). A human ortholog of archaeal DNA repair protein Hef is defective in
Fanconi anemia complementation group M. Nat Genet.

[B50] Meijer GA (2007). The 13th Fanconi anemia gene identified: FANCI - Importance of the
‘Fanconi anemia pathway’ for cellular oncology. Cell Oncol.

[B51] Michl J, Zimmer J, Tarsounas M (2016). Interplay between Fanconi anemia and homologous recombination pathways
in genome integrity. EMBO J.

[B52] Mullen JR, Kaliraman V, Ibrahim SS, Brill SJ (2001). Requirement for three novel protein complexes in the absence of the
Sgs1 DNA helicase in Saccharomyces cerevisiae. Genetics.

[B53] Munoz IM, Hain K, Declais AC, Gardiner M, Toh GW, Sanchez-Pulido L, Heuckmann JM, Toth R, Macartney T, Eppink B (2009). Coordination of structure-specific nucleases by human SLX4/BTBD12 is
required for DNA repair. Mol Cell.

[B54] Myers KC, Bleesing JJ, Davies SM, Zhang X, Martin LJ, Mueller R, Harris RE, Filipovich AH, Kovacic MB, Wells SI (2011). Impaired immune function in children with Fanconi
anaemia. Br J Haematol.

[B55] Nguyen TV, Riou L, Aoufouchi S, Rosselli F (2014). Fanca deficiency reduces A/T transitions in somatic hypermutation and
alters class switch recombination junctions in mouse B cells. J Exp Med.

[B56] Novotna B, Goetz P, Surkova NI (1979). Effects of alkylating agents on lymphocytes from controls and from
patients with Fanconi's anemia. Studies of sister chromatid exchanges, chromosome
aberrations, and kinetics of cell division. Hum Genet.

[B57] Pagano G, Manini P, Bagchi D (2003). Oxidative stress-related mechanisms are associated with xenobiotics
exerting excess toxicity to Fanconi anemia cells. Environ Health Perspect.

[B58] Pagano G, Talamanca AA, Castello G, Pallardo FV, Zatterale A, Degan P (2012). Oxidative stress in Fanconi anaemia: From cells and molecules towards
prospects in clinical management. Biol Chem.

[B59] Pang Q, Christianson TA, Keeble W, Diaz J, Faulkner GR, Reifsteck C, Olson S, Bagby GC (2001). The Fanconi anemia complementation group C gene product: Structural
evidence of multifunctionality. Blood.

[B60] Park JY, Virts EL, Jankowska A, Wiek C, Othman M, Chakraborty SC, Vance GH, Alkuraya FS, Hanenberg H, Andreassen PR (2016). Complementation of hypersensitivity to DNA interstrand crosslinking
agents demonstrates that XRCC2 is a Fanconi anaemia gene. J Med Genet.

[B61] Parker L, dos Santos C, Buchwald M (1998). The delta327 mutation in the Fanconi anemia group C gene generates a
novel transcript lacking the first two coding exons. Hum Mutat.

[B62] Parodi A, Kalli F, Svahn J, Stroppiana G, De Rocco D, Terranova P, Dufour C, Fenoglio D, Cappelli E (2015). Impaired immune response to *Candida albicans* in cells
from Fanconi anemia patients. Cytokine.

[B63] Pinto FO, Leblanc T, Chamousset D, Le Roux G, Brethon B, Cassinat B, Larghero J, de Villartay JP, Stoppa-Lyonnet D, Baruchel A (2009). Diagnosis of Fanconi anemia in patients with bone marrow
failure. Haematologica.

[B64] Pronk JC, Gibson RA, Savoia A, Wijker M, Morgan NV, Melchionda S, Ford D, Temtamy S, Ortega JJ, Jansen S (1995). Localisation of the Fanconi anaemia complementation group A gene to
chromosome 16q24.3. Nat Genet.

[B65] Reid S, Schindler D, Hanenberg H, Barker K, Hanks S, Kalb R, Neveling K, Kelly P, Seal S, Freund M (2007). Biallelic mutations in PALB2 cause Fanconi anemia subtype FA-N and
predispose to childhood cancer. Nat Genet.

[B66] Rosenberg PS, Tamary H, Alter BP (2011). How high are carrier frequencies of rare recessive syndromes?
Contemporary estimates for Fanconi Anemia in the United States and
Israel. Am J Med Genet A.

[B67] Rosselli F, Sanceau J, Gluckman E, Wietzerbin J, Moustacchi E (1994). Abnormal lymphokine production: A novel feature of the genetic disease
Fanconi anemia. II. In vitro and in vivo spontaneous overproduction of tumor
necrosis factor alpha. Blood.

[B68] Sawyer SL, Tian L, Kahkonen M, Schwartzentruber J, Kircher M, Consortium FC, Majewski J, Dyment DA, Innes AM, University of Washington Centre for Mendelian Genomics (2015). Biallelic mutations in BRCA1 cause a new Fanconi anemia
subtype. Cancer Discovery.

[B69] Sims AE, Spiteri E, Sims RJ, Arita AG, Lach FP, Landers T, Wurm M, Freund M, Neveling K, Hanenberg H (2007). FANCI is a second monoubiquitinated member of the Fanconi anemia
pathway. Nat Struct Mol Biol.

[B70] Singh TR, Bakker ST, Agarwal S, Jansen M, Grassman E, Godthelp BC, Ali AM, Du CH, Rooimans MA, Fan Q (2009). Impaired FANCD2 monoubiquitination and hypersensitivity to
camptothecin uniquely characterize Fanconi anemia complementation group
M. Blood.

[B71] Smogorzewska A, Matsuoka S, Vinciguerra P, McDonald ER, Hurov KE, Luo J, Ballif BA, Gygi SP, Hofmann K, D'Andrea AD (2007). Identification of the FANCI protein, a monoubiquitinated FANCD2
paralog required for DNA repair. Cell.

[B72] Somyajit K, Subramanya S, Nagaraju G (2012). Distinct roles of FANCO/RAD51C protein in DNA damage signaling and
repair: Implications for Fanconi anemia and breast cancer
susceptibility. J Biol Chem.

[B73] Stoepker C, Hain K, Schuster B, Hilhorst-Hofstee Y, Rooimans MA, Steltenpool J, Oostra AB, Eirich K, Korthof ET, Nieuwint AW (2011). SLX4, a coordinator of structure-specific endonucleases, is mutated in
a new Fanconi anemia subtype. Nat Genet.

[B74] Strathdee CA, Duncan AM, Buchwald M (1992a). Evidence for at least four Fanconi anaemia genes including FACC on
chromosome 9. Nat Genet.

[B75] Strathdee CA, Gavish H, Shannon WR, Buchwald M (1992b). Cloning of cDNAs for Fanconi's anaemia by functional
complementation. Nature.

[B76] Sumpter R, Sirasanagandla S, Fernandez AF, Wei Y, Dong X, Franco L, Zou Z, Marchal C, Lee MY, Clapp DW (2016). Fanconi anemia proteins function in mitophagy and
immunity. Cell.

[B77] Svendsen JM, Smogorzewska A, Sowa ME, O'Connell BC, Gygi SP, Elledge SJ, Harper JW (2009). Mammalian BTBD12/SLX4 assembles a Holliday junction resolvase and is
required for DNA repair. Cell.

[B78] Timmers C, Taniguchi T, Hejna J, Reifsteck C, Lucas L, Bruun D, Thayer M, Cox B, Olson S, D'Andrea AD (2001). Positional cloning of a novel Fanconi anemia gene,
FANCD2. Mol Cell.

[B79] Vaz F, Hanenberg H, Schuster B, Barker K, Wiek C, Erven V, Neveling K, Endt D, Kesterton I, Autore F (2010). Mutation of the RAD51C gene in a Fanconi anemia-like
disorder. Nat Genet.

[B80] Waisfisz Q, Saar K, Morgan NV, Altay C, Leegwater PA, de Winter JP, Komatsu K, Evans GR, Wegner RD, Reis A (1999). The Fanconi anemia group E gene, FANCE, maps to chromosome
6p. Am J Hum Genet.

[B81] Wang W (2007). Emergence of a DNA-damage response network consisting of Fanconi
anaemia and BRCA proteins. Nat Rev Genet.

[B82] Whitney M, Thayer M, Reifsteck C, Olson S, Smith L, Jakobs PM, Leach R, Naylor S, Joenje H, Grompe M (1995). Microcell mediated chromosome transfer maps the Fanconi anaemia group
D gene to chromosome 3p. Nat Genet.

[B83] Wu HI, Brown JA, Dorie MJ, Lazzeroni L, Brown JM (2004). Genome-wide identification of genes conferring resistance to the
anticancer agents cisplatin, oxaliplatin, and mitomycin C. Cancer Res.

[B84] Xia B, Sheng Q, Nakanishi K, Ohashi A, Wu J, Christ N, Liu X, Jasin M, Couch FJ, Livingston DM (2006). Control of BRCA2 cellular and clinical functions by a nuclear partner,
PALB2. Mol Cell.

[B85] Xia B, Dorsman JC, Ameziane N, de Vries Y, Rooimans MA, Sheng Q, Pals G, Errami A, Gluckman E, Llera J (2007). Fanconi anemia is associated with a defect in the BRCA2 partner
PALB2. Nat Genet.

[B86] Zakrzewski S, Sperling K (1980). Genetic heterogeneity of Fanconi's anemia demonstrated by somatic cell
hybrids. Hum Genet.

[B87] Zanier R, Briot D, Dugas du Villard JA, Sarasin A, Rosselli F (2004). Fanconi anemia C gene product regulates expression of genes involved
in differentiation and inflammation. Oncogene.

[B88] Zhang QH, Ye M, Wu XY, Ren SX, Zhao M, Zhao CJ, Fu G, Shen Y, Fan HY, Lu G (2000). Cloning and functional analysis of cDNAs with open reading frames for
300 previously undefined genes expressed in CD34+ hematopoietic stem/progenitor
cells. Genome Res.

